# Supplementary figures and images for: A prosthesis utilizing natural vestibular encoding strategies improves sensorimotor performance in monkeys
Source: PLoS Biol. 2022 Sep 14;20(9):e3001798. doi: 10.1371/journal.pbio.3001798 (PMC9473632; doi:10.1371/journal.pbio.3001798)

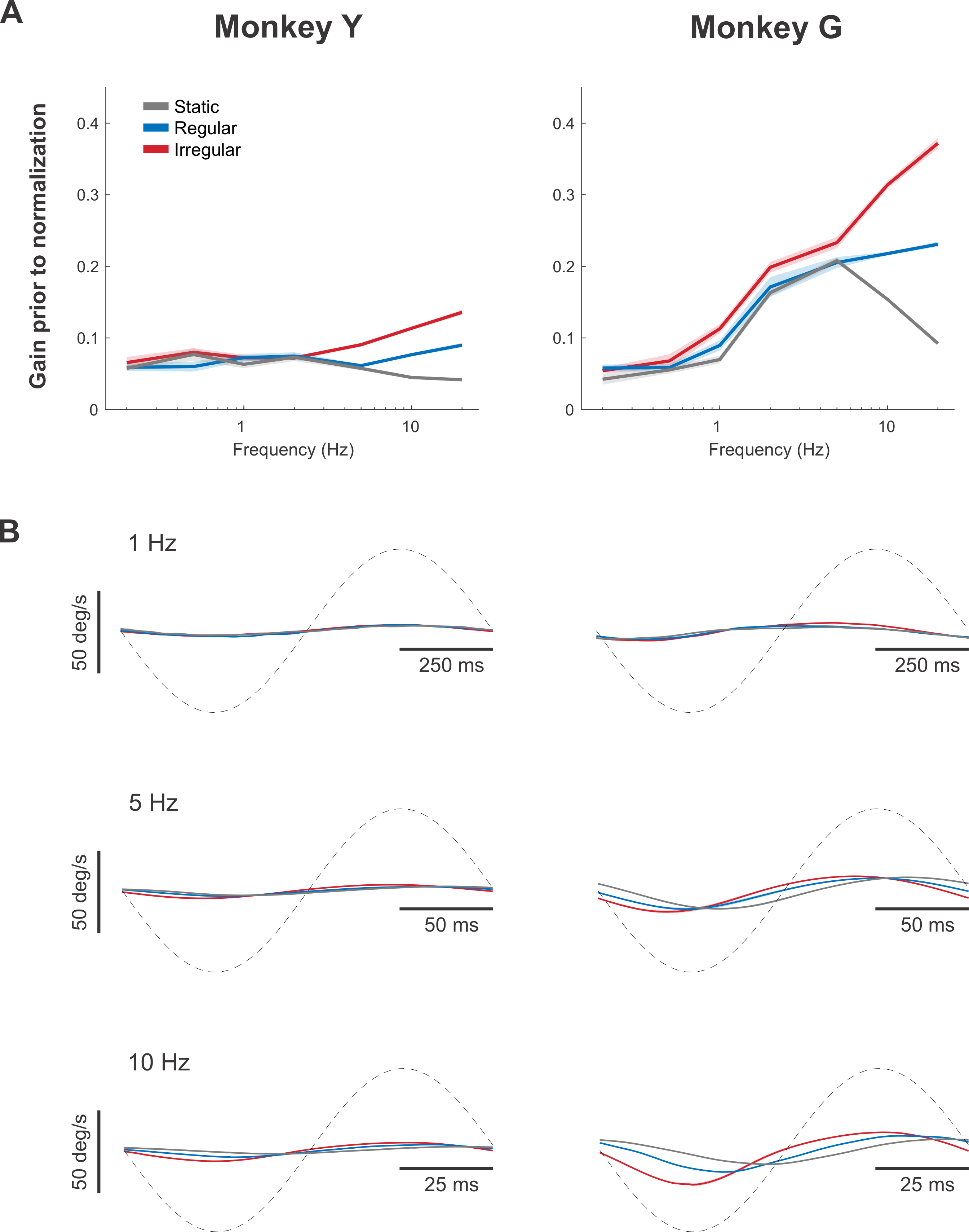

Supplement: S1 Fig — (A) VOR gains prior to normalization across natural frequency range (0.2–20 Hz). Note that the figure shows the same data as Fig 2B but the gains are displayed on the absolute scale (not normalized). (B) Example VOR traces prior to normalization. Dashed lines indicate inverted, virtual head velocity. The shaded areas indicate SEM. Red, blue, and gray refer to the irregular, regular, and static mappings, respectively. Data underlying this figure can be found at https://doi.org/10.5281/zenodo.6338639. (TIF) [file pbio.3001798.s001.tif]

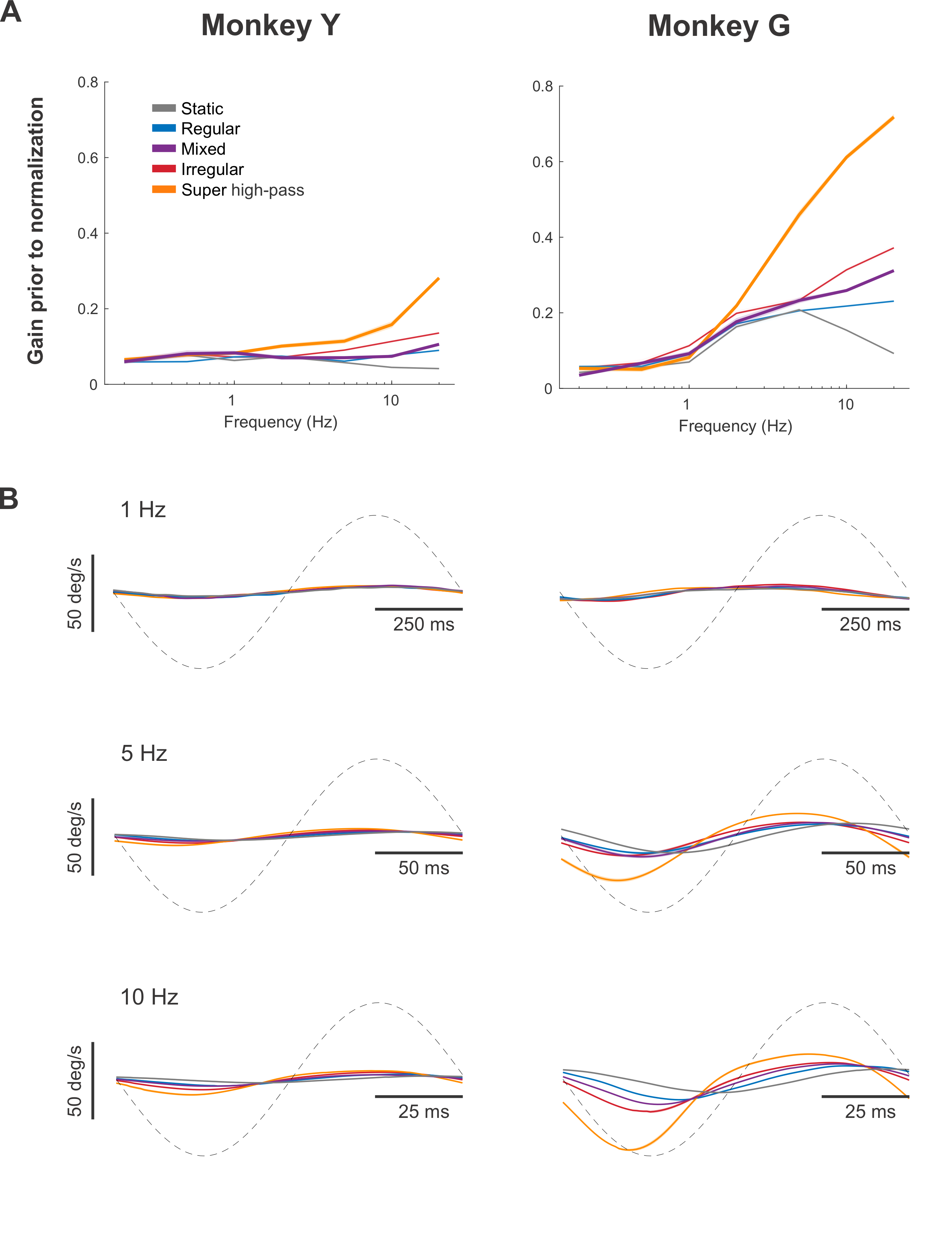

Supplement: S2 Fig — (A) VOR gains prior to normalization across natural frequency range (0.2–20 Hz). Note that the figure shows the same data as Fig 3C but the gains are displayed on the absolute scale (not normalized). (B) Example VOR traces prior to normalization. Dashed lines indicate the inverted, virtual head velocity. The shaded areas indicate SEM. Yellow, red, purple, blue, and gray refer to the super high-pass, irregular, mixed, regular, and static mappings, respectively. Data underlying this figure can be found at https://doi.org/10.5281/zenodo.6338639. (TIF) [file pbio.3001798.s002.tif]

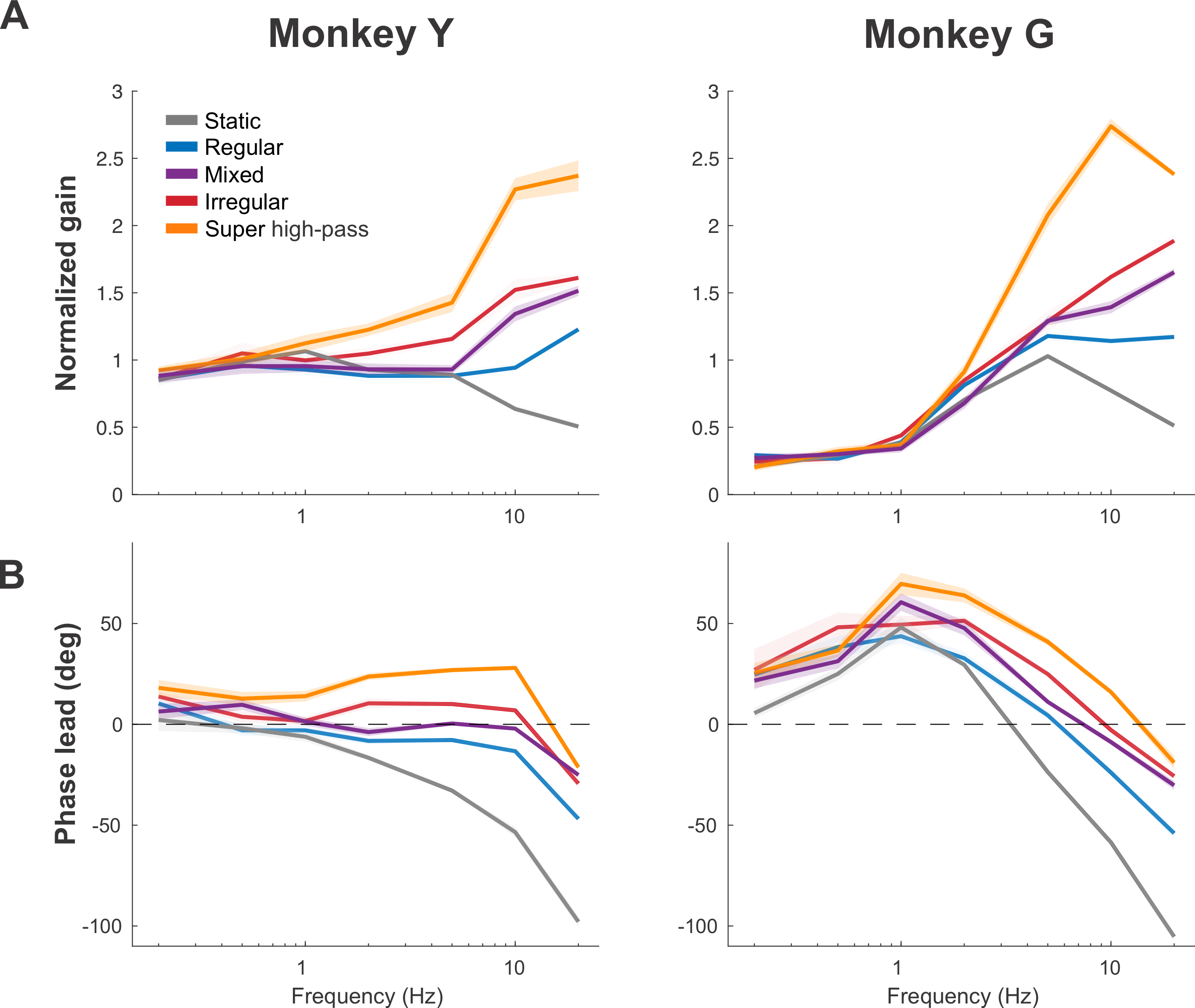

Supplement: S3 Fig — (A) Normalized VOR gains across natural frequency range (0.2–20 Hz), using the same normalization reference as Fig 2. Note the reduction in gain at 20 Hz due to saturation. (B) The phase response of the VOR. Note the similarity to the phase response in the X1 condition in Fig 3D. The shaded area indicated the SEM. Yellow, red, purple, blue, and gray refer to the super high-pass, irregular, mixed, regular, and static mappings, respectively. Data underlying this figure can be found at https://doi.org/10.5281/zenodo.6338639. (TIF) [file pbio.3001798.s003.tif]

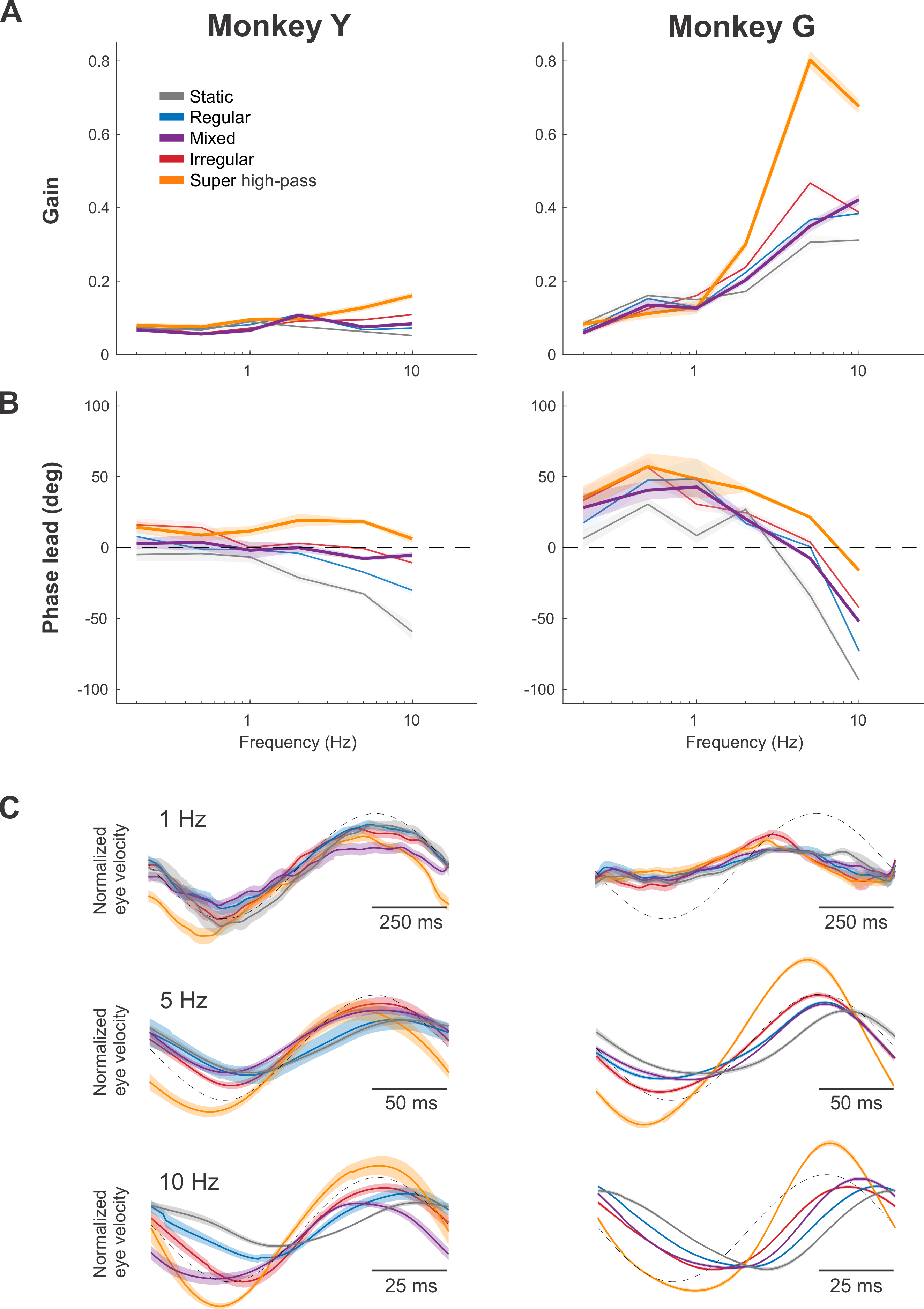

Supplement: S4 Fig — (A) VOR gains across the frequency range (0.2–10 Hz). (B) The phase response of the VOR. (C) Example VOR traces. Dashed lines indicate inverted head velocity. There was a significant increase in the VOR gain mainly over the 5–10 Hz range for Monkey G (p < 0.05, Bonferroni corrected) but not for Monkey Y. There was also a significant increase in phase lead mainly over 5–10 Hz range for both monkeys (p < 0.05, Bonferroni corrected). There was no significant difference in the gain and phase responses for early and late cycles in both monkeys. Yellow, red, purple, blue, and gray refer to the super high-pass, irregular, mixed, regular, and static mappings, respectively. Data underlying this figure can be found at https://doi.org/10.5281/zenodo.6338639. (TIF) [file pbio.3001798.s004.tif]

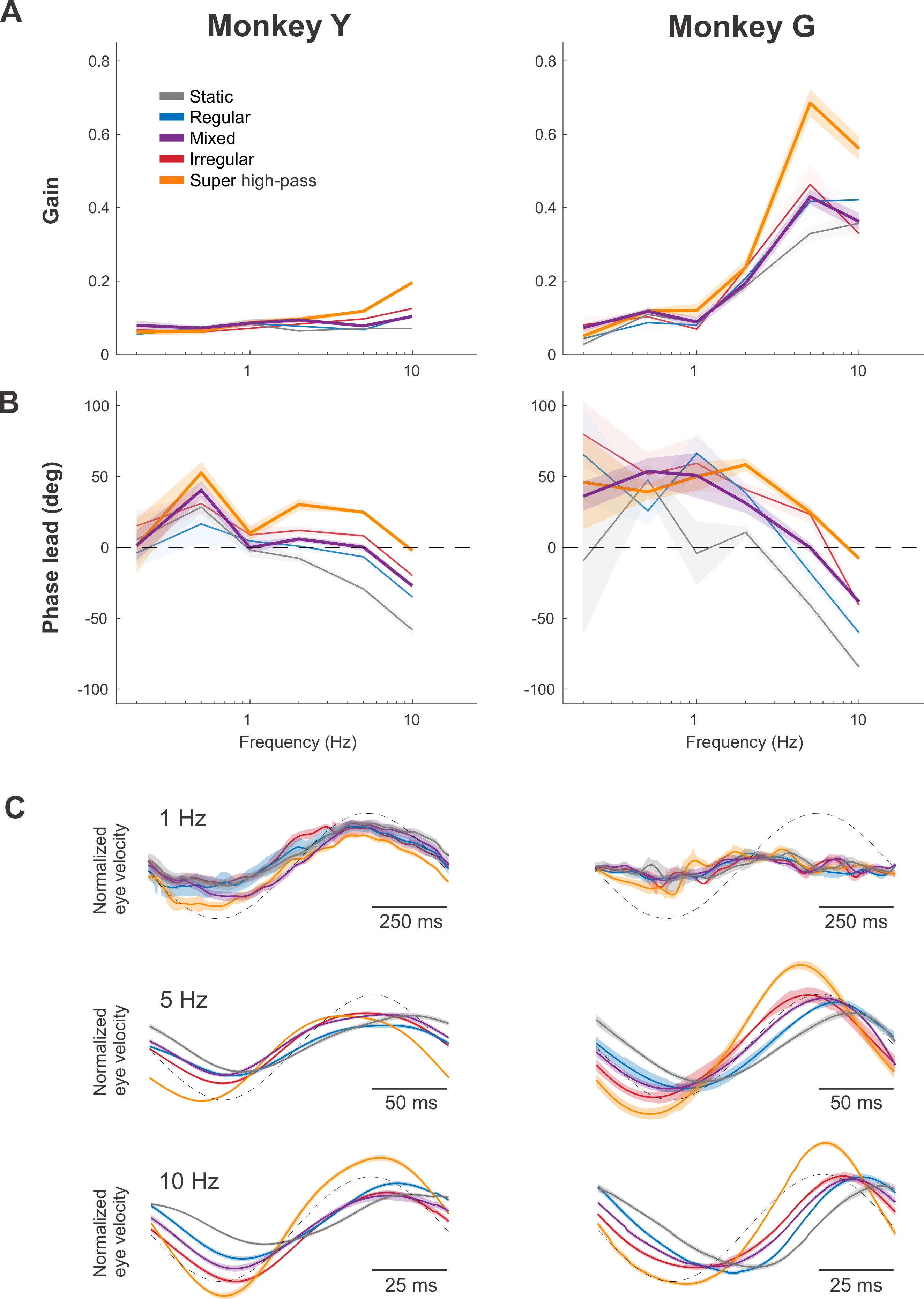

Supplement: S5 Fig — (A) VOR gains across the frequency range (0.2–10 Hz). (B) The phase response of the VOR. (C) Example VOR traces. Dashed line indicated the inverted head velocity. There was a significant increase in the VOR gain mainly over the 5–10 Hz range for Monkey G (p < 0.05, Bonferroni corrected) and at 10 Hz for Monkey Y (p < 0.05, Bonferroni corrected). There was also a significant increase in phase lead mainly over 5–10 Hz range for both monkeys (p < 0.05, Bonferroni corrected). There was no significant difference in the gain and phase responses for early and late cycles in both monkeys. Yellow, red, purple, blue, and gray refer to the super high-pass, irregular, mixed, regular, and static mappings, respectively. Data underlying this figure can be found at https://doi.org/10.5281/zenodo.6338639. (TIF) [file pbio.3001798.s005.tif]

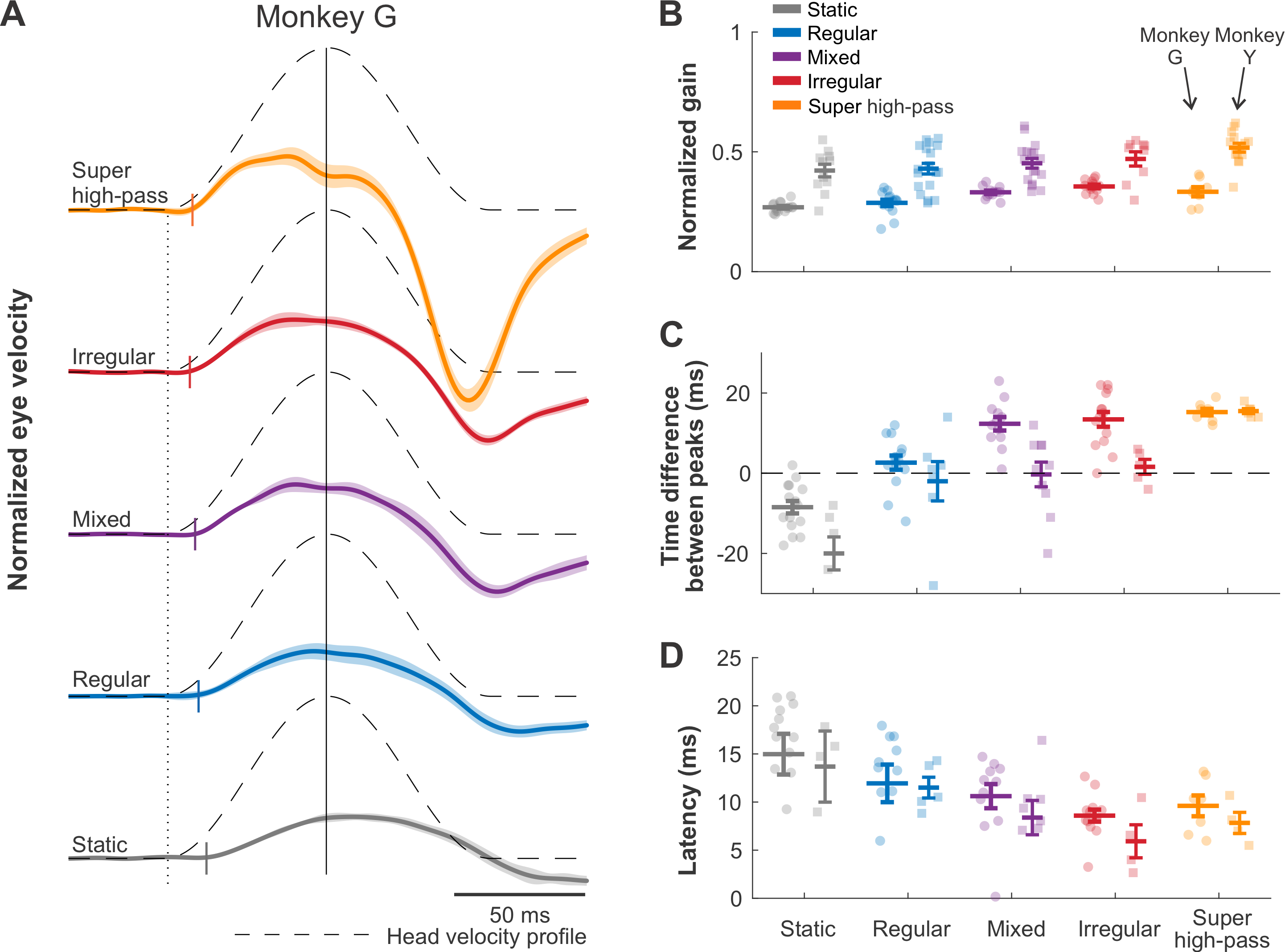

Supplement: S6 Fig — (A) Traces of the evoked eye movements for Monkey G during transient head movements (off-direction), normalized as in Fig 4. Dashed lines indicate the inverted head velocity. Dotted vertical line indicates the start of the head movements. Solid vertical line denotes the peak of the head movements. Short colored vertical lines indicated the estimated onset of the evoked eye movement. (B, C, and D) Quantification of the traces in (A) for normalized gain [using the same normalization reference as in Fig 4], time difference between eye and head velocity peaks, and onset latency, respectively. Results from Monkey G and Monkey Y are plotted on the left and on the right, respectively. Error bars indicate the SEM. Yellow, red, purple, blue, and gray refer to the super high-pass, irregular, mixed, regular, and static mappings, respectively. Similar to the on-direction results, the biomimetic regular and mixed mappings evoked a peak VOR response that was well aligned with the peak head velocity (not significantly different from 0 ms except for Monkey G mixed mapping, which showed a significant but small lead of 12.3 ± 1.7 ms, p < 0.01, Bonferroni corrected). In contrast, the static mapping resulted in the VOR peak with a significant delay (p < 0.001 for Monkey G, not significant for Monkey Y, Bonferroni corrected), while the VOR peaks evoked by irregular and super high-pass mappings actually led the stimulus (p < 0.05 for Monkey G, not significant for Monkey Y). Data underlying this figure can be found at https://doi.org/10.5281/zenodo.6338639. (TIF) [file pbio.3001798.s006.tif]

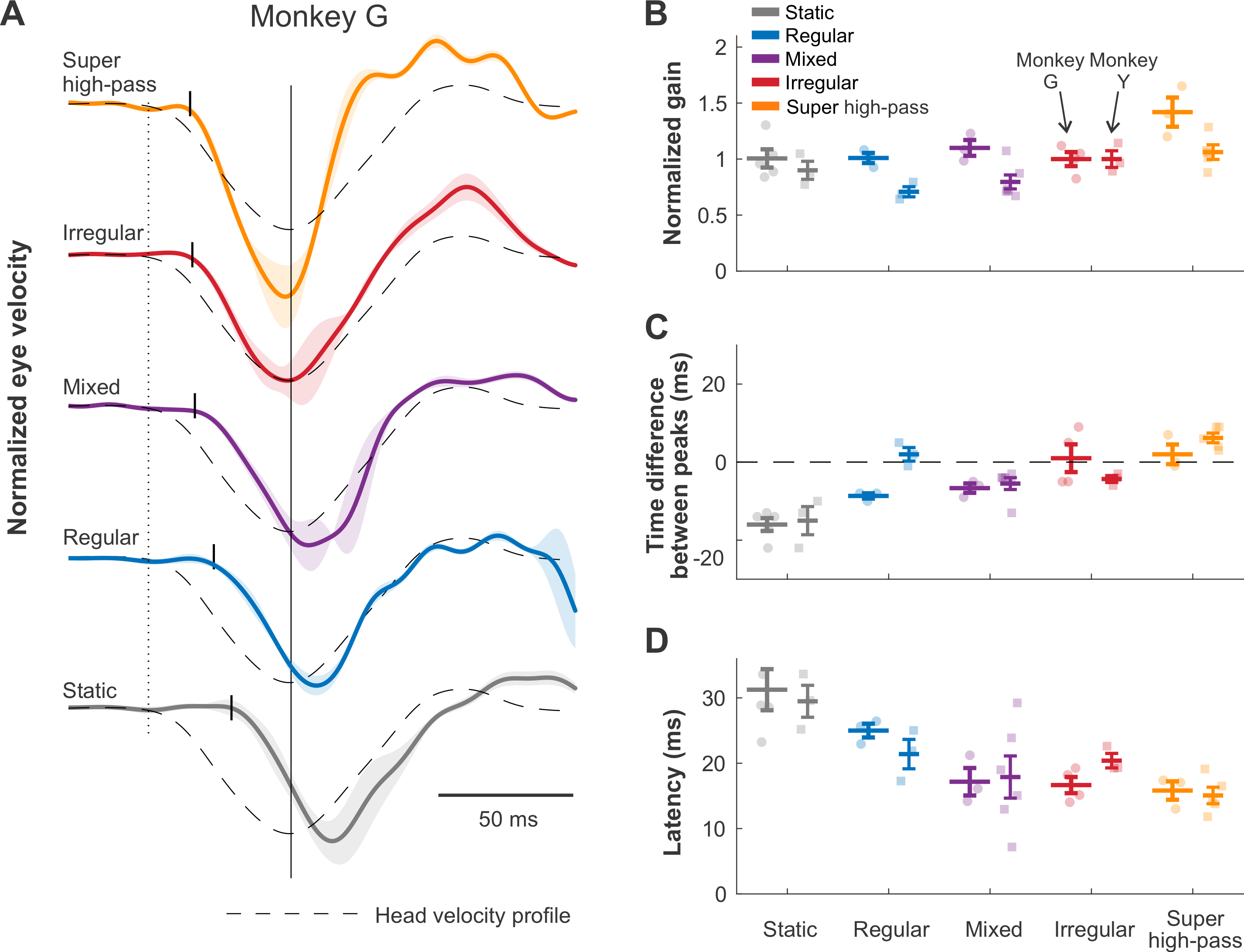

Supplement: S7 Fig — (A) Traces of the evoked eye movements for Monkey G during physical transient head movements (on-direction), normalized as in Fig 4. Dashed lines indicate the inverted head velocity, scaled to facilitate timing comparison. Dotted vertical line indicates the start of the head movements. Solid vertical line denotes the peak of the head movements. Short vertical lines indicated the estimated onset of the evoked eye movement. (B, C, and D) Quantification of the traces in (A) for normalized gain, time difference between eye and head velocity peaks, and onset latency, respectively. Results from Monkey G and Monkey Y are plotted on the left and on the right, respectively. Error bars indicate the SEM. Yellow, red, purple, blue, and gray refer to the super high-pass, irregular, mixed, regular, and static mappings, respectively. Data underlying this figure can be found at https://doi.org/10.5281/zenodo.6338639. (TIF) [file pbio.3001798.s007.tif]

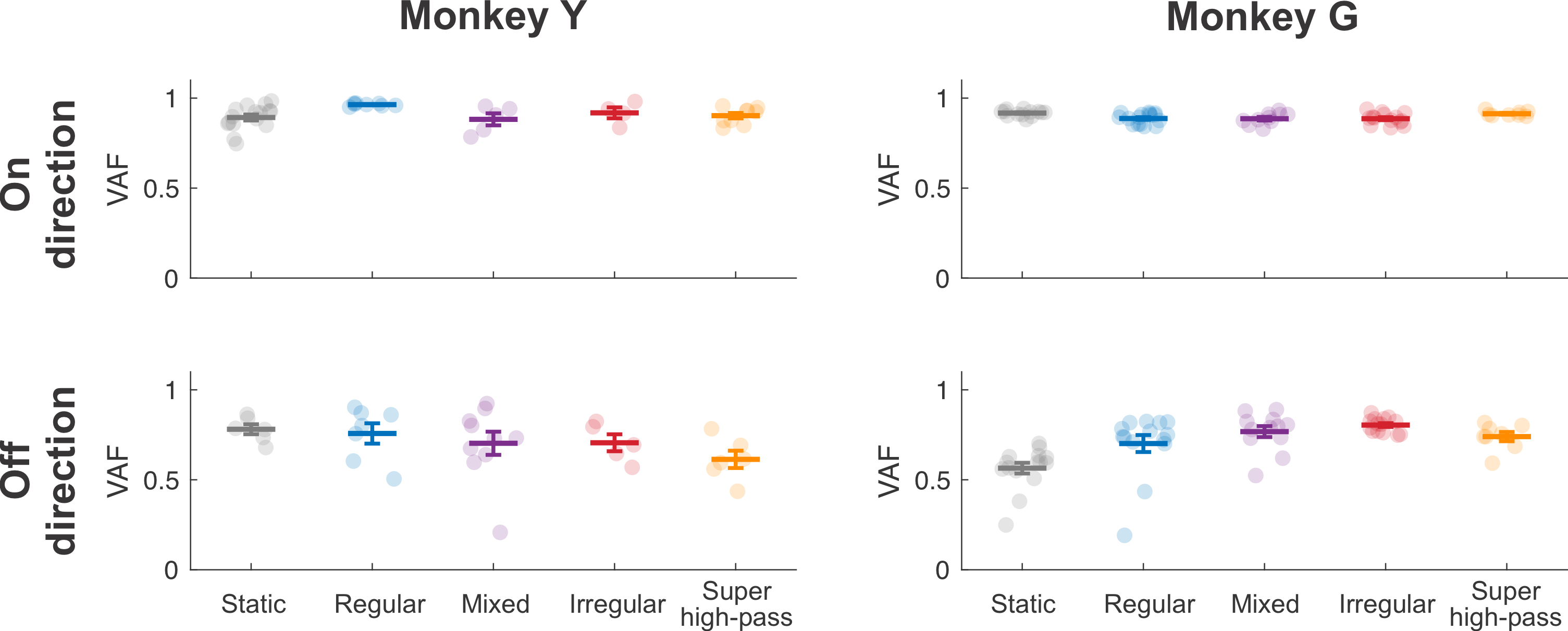

Supplement: S8 Fig — The VAFs are plotted for each trial of the head movements in the on (top row) and off (bottom rows) directions for Monkey Y (left column) and Monkey G (right column). Error bars indicate the SEM. Data underlying this figure can be found at https://doi.org/10.5281/zenodo.6338639. (TIF) [file pbio.3001798.s008.tif]

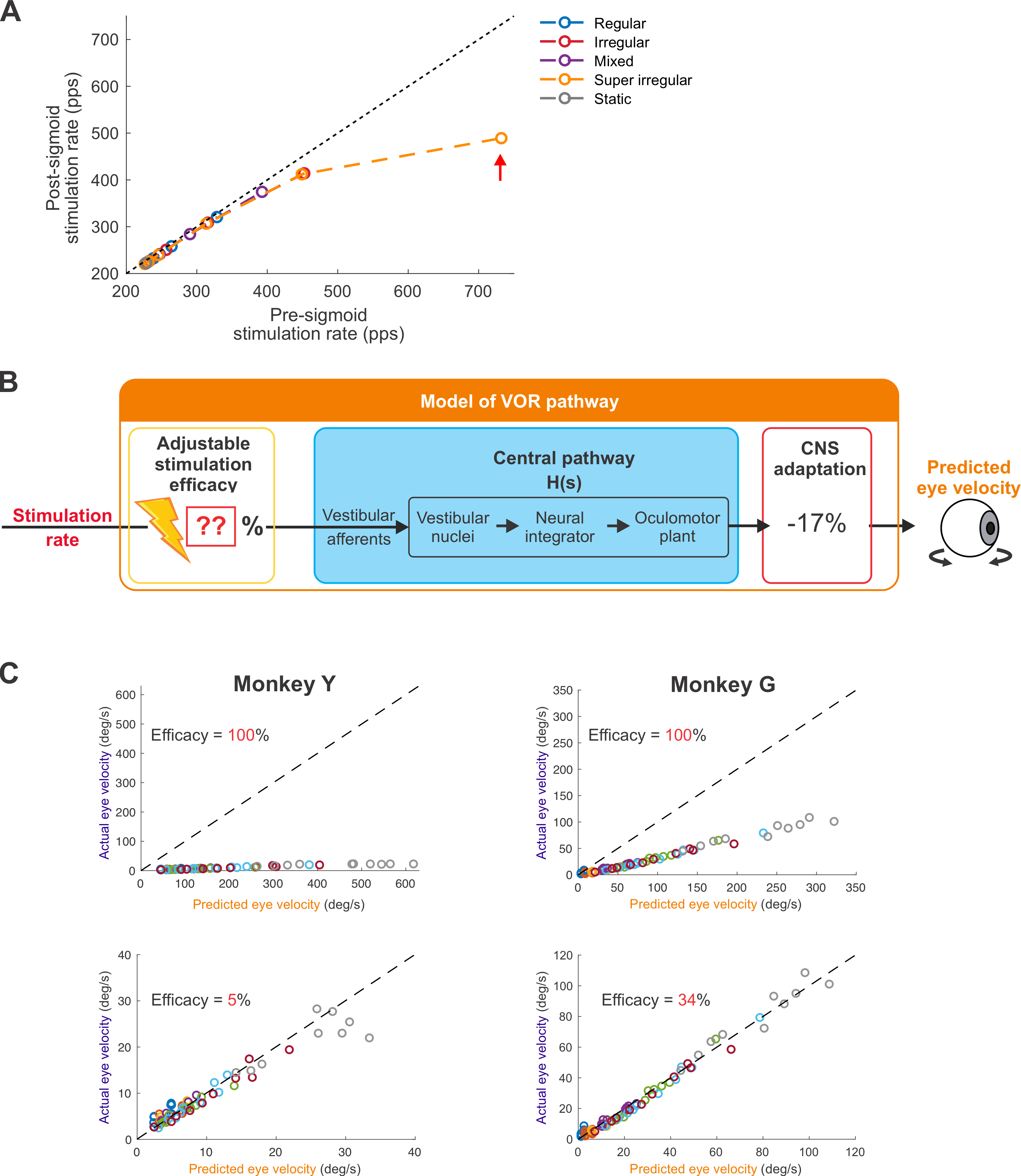

Supplement: S9 Fig — (A) Post-sigmoid stimulation rate plotted as a function of pre-sigmoid stimulation rate. The red arrow points to the data point from the super high-pass 2× gain mapping at 20 Hz, which showed large deviation from linearity. (B) Similar to the model in Fig 6C but with the addition of the CNS adaptation (17%; [18]). (C) Plots of the actual eye velocity and the predicted eye velocity from the model in (B) using 100% stimulation efficacy (top) and the lower stimulation efficacy that best fit the data (bottom). The dashed line is the unity line. Data underlying this figure can be found at https://doi.org/10.5281/zenodo.6338639. (TIF) [file pbio.3001798.s009.tif]
