# Supplementary material for: A prosthesis utilizing natural vestibular encoding strategies improves sensorimotor performance in monkeys
Source: PLoS Biol. 2022 Sep 14;20(9):e3001798. doi: 10.1371/journal.pbio.3001798 (PMC9473632; doi:10.1371/journal.pbio.3001798)
Supplement: Attachment — Submitted filename: 2022 06 27VOR_paper PlosBio Review Responses.docx [file pbio.3001798.s010.docx]

­­­­Dear Dr Cullen,

Thank you for submitting your manuscript "Restoring vestibular afferent dynamics enhances sensorimotor performance: implications for vestibular prostheses" for consideration as a Research Article at PLOS Biology. Your manuscript has been evaluated by the PLOS Biology editors, an Academic Editor with relevant expertise, and by several independent reviewers.

We thank the PLOS Biology editors, the Academic Editor, and the reviewers for their comments and suggestions. We have provided a point-by-point response to address each of the editor’s and reviewer’s specific comments below. In particular, we have made major revisions to the manuscript that include new data on prosthetic stimulation patterns evoked by actual physical head movements (new figures S4, S5. And S7), revised the discussion to more clearly address the low VOR gains and the gain difference between animals, and expanded the discussion on the implications of our results to be more accessible to the broad scientific readership at PLOS Biology.

In light of the reviews (below), we will not be able to accept the current version of the manuscript, but we would welcome re-submission of a much-revised version that takes into account all of the reviewers’ comments. In considering the reviewer feedback, we particularly note that all of the Reviewers expressed concerns with the low VOR-evoked gain you observe and Reviewer 1 raised some additional concerns with your virtual head motion design. The Academic Editor agreed that a particular concern is the low gain of the prothesis based VOR and that this is further complicated by the gain difference you see between the 2 animals. They also suggested that a potential explanation for the low gains, might, in fact, be related to the study reliance on direct stimulation rather than stimulation reflecting natural head movements.

In any revision we would therefore be particularly looking to see how you address the concerns related to gain levels and head motion. Along these lines, if possible, we ask that you add data on prosthetic stimulation patterns reflecting natural head movements. If you do not have such data and the experimental animals are no longer available for further assessment, we would be looking for a convincing argument as to why a consideration of natural head movements is not needed to appreciate that relevance of “nature-like” stimulation patterns. This is particularly important given the potential usage of your findings for prostheses design and would be a consideration for any final decision on your study. Additionally, we would expect to see a substantial revamping of the study presentation in any revision to PLOS Biology to ensure your study is accessible to our broad scientific readership. This should also include some expansion to the discussion to further touch on the broader implications of your work beyond the vestibular system and how this fits in with evidence from other domains – something you only touch on very briefly at the moment.

We thank the editors for their suggestions on the revision. We have addressed each of the comments below.

To directly address the reviewers’ main concerns, we have added to the revised manuscript new data on prosthetic stimulation patterns evoked by actual physical head movements. Specifically, for the same two animals, we now show the eye movements evoked by prosthesis stimulation encoding real-time head velocity that was detected by the gyroscope module of the prosthesis. These additional data are shown in new figures: figs. S4, S5, and S7.

As can be seen in the revised manuscript, the trends are the same for the virtual-rotation and physical-rotation conditions. Specifically:

- For sinusoidal stimuli (Fig. 3 vs. figs. S4-5; dark and light conditions, respectively), the gains of the biomimetic mappings remained more robust, and their phase responses were closer to zero while the static mapping produced smaller gain and increasing phase lag. In addition, the super high-pass mapping, again, produced unfavorable increasing gain and phase lead. Additionally, as now described in the revised results, higher gains were indeed observed in the physical-rotation conditions at higher stimulation frequencies (>5Hz).
- Likewise transient VOR responses during physical-rotation condition were also similar to the results during virtual head motion (Fig. 4 vs. fig. S7). Specifically, the static mapping yielded delayed response compared to the timing of the head motion, while more biomimetic mappings yielded responses that were more closely aligned with the head motion, which resulted in more stable gaze. As in the case of higher frequency sinusoidal stimulation, higher gains were observed in the physical-rotation versus virtual-rotation transient condition, for both animals.

Thus, together these results indicate that the observed improvement in VOR accuracy was consistent across movement conditions and that the higher VOR gains could be achieved during physical head rotations.

We have also expanded the revised Discussion to more clearly emphasize that prosthesis-evoked VOR gains are not only generally low, but also variable across subjects in both humans (Boutros et al., 2019; Fornos et al., 2014, Phillips et al., 2015) and monkeys (Dai et al., 2011; Boutros et al. 2019). Notably, prior studies using comparable protheses have shown >5 fold differences in gain across individual subjects (e.g., Phillips et al., 2015; Boutros et al. 2019), depending on frequency. These differences are likely, at least in part, due to variability in the placement of the electrodes targeting the ampullae (Hedjoudje et al., 2019), which is an ongoing focus for optimization. In this context, the similar trends observed for the two monkeys despite the gain difference strengthen the implications of the current study, since all implanted patients would likely benefit from the inclusion of biomimetic encoding regardless of the exact placement of their implants. Additionally, our present results established that VOR gains can be improved by increasing the mapping gains (Fig. 3E, similar trends for both animals). In this context, our results predict that the maximum stimulation rate currently used in humans (Boutros 2019, Chow, 2019) could be extended without saturating the afferent firing. This would allow for increasing the mapping gain while also maintaining the dynamic range.

Finally, we have revised the discussion to broaden the current results and make the implications of our study more accessible to the broad scientific readership at PLOS Biology.

- Specifically, our present findings serve to extend a growing body of literature showing improved functional performance from biomimetic sensory prosthesis designs (e.g., enhanced speech intelligibility for cochlear implants, Fumero et al., 2021; improved manual dexterity for limb prostheses that include tactile feedback, Valle et al. 2018, George et al., 2019, Osborn et al., 2018).
- Additionally, our present findings further show that accounting for the specific afferent input to a well-defined sensorimotor pathway can optimize functional performance (i.e., VOR). Similar to the vestibular system, other sensory systems are characterized by heterogeneity in the afferent inputs mediating different behavioral and perceptual outcomes. For example, in the somatosensory system, slow adapting mechanoreceptors detect sustained force and are thought to better aid in adjusting grip force, while fast adapting receptors detect changes in force and are thought to contribute to the perception of vibration (Abraira & Ginty, 2013). As now noted in the revised discussion, we suggest that the development of prostheses that can account for such pathway specific heterogeneities will be essential to improving functional outcomes across sensory systems.

Please note that we cannot make any decision about publication until we have seen the revised manuscript and your response to the reviewers' comments. Your revised manuscript is also likely to be sent for further evaluation by the reviewers.

We expect to receive your revised manuscript within 3 months.

Please email us (plosbiology@plos.org) if you have any questions or concerns, or would like to request an extension. At this stage, your manuscript remains formally under active consideration at our journal; please notify us by email if you do not intend to submit a revision so that we may end consideration of the manuscript at PLOS Biology.

**IMPORTANT - SUBMITTING YOUR REVISION**

Your revisions should address the specific points made by each reviewer. Please submit the following files along with your revised manuscript:

1. A 'Response to Reviewers' file - this should detail your responses to the editorial requests, present a point-by-point response to all of the reviewers' comments, and indicate the changes made to the manuscript.
*NOTE: In your point by point response to the reviewers, please provide the full context of each review. Do not selectively quote paragraphs or sentences to reply to. The entire set of reviewer comments should be present in full and each specific point should be responded to individually, point by point.
You should also cite any additional relevant literature that has been published since the original submission and mention any additional citations in your response.

2. In addition to a clean copy of the manuscript, please also upload a 'track-changes' version of your manuscript that specifies the edits made. This should be uploaded as a "Related" file type.

*Re-submission Checklist*
When you are ready to resubmit your revised manuscript, please refer to this re-submission checklist: https://plos.io/Biology_Checklist

To submit a revised version of your manuscript, please go to https://www.editorialmanager.com/pbiology/ and log in as an Author. Click the link labelled 'Submissions Needing Revision' where you will find your submission record.

Please make sure to read the following important policies and guidelines while preparing your revision:

*Published Peer Review*
Please note while forming your response, if your article is accepted, you may have the opportunity to make the peer review history publicly available. The record will include editor decision letters (with reviews) and your responses to reviewer comments. If eligible, we will contact you to opt in or out. Please see here for more details:
https://blogs.plos.org/plos/2019/05/plos-journals-now-open-for-published-peer-review/

*PLOS Data Policy*
Please note that as a condition of publication PLOS' data policy (http://journals.plos.org/plosbiology/s/data-availability) requires that you make available all data used to draw the conclusions arrived at in your manuscript. If you have not already done so, you must include any data used in your manuscript either in appropriate repositories, within the body of the manuscript, or as supporting information (N.B. this includes any numerical values that were used to generate graphs, histograms etc.). For an example see here: http://www.plosbiology.org/article/info%3Adoi%2F10.1371%2Fjournal.pbio.1001908#s5

*Blot and Gel Data Policy*
We require the original, uncropped and minimally adjusted images supporting all blot and gel results reported in an article's figures or Supporting Information files. We will require these files before a manuscript can be accepted so please prepare them now, if you have not already uploaded them. Please carefully read our guidelines for how to prepare and upload this data: https://journals.plos.org/plosbiology/s/figures#loc-blot-and-gel-reporting-requirements

*Protocols deposition*
To enhance the reproducibility of your results, we recommend that if applicable you deposit your laboratory protocols in protocols.io, where a protocol can be assigned its own identifier (DOI) such that it can be cited independently in the future. Additionally, PLOS ONE offers an option for publishing peer-reviewed Lab Protocol articles, which describe protocols hosted on protocols.io. Read more information on sharing protocols at https://plos.org/protocols?utm_medium=editorial-email&utm_source=authorletters&utm_campaign=protocols

Thank you again for your submission to our journal. We hope that our editorial process has been constructive thus far, and we welcome your feedback at any time. Please don't hesitate to contact us if you have any questions or comments.

Sincerely,

Kris


Kris Dickson
Neurosciences Senior Editor/Section Manager
PLOS Biology
kdickson@plos.org

*****************************************************
REVIEWS:

Reviewer's Responses to Questions

PLOS authors have the option to publish the peer review history of their article ([what does this mean?](https://journals.plos.org/plosbiology/s/editorial-and-peer-review-process#loc-peer-review-history)). If published, this will include your full peer review and any attached files.


If you choose “no”, your identity will remain anonymous but your review may still be made public.

**Reviewer #1:**

This is an extremely interesting and provocative paper. It is well and clearly written and the figures are superb. That is this manuscript certainly deserves to be published. I have only one major remark concerning the choice of recording the VOR in head fixed monkeys. The rest of my comments, apart from typos, concerned the "pedagogic" aspects of the introduction and result sections. This paper deserves a large audience and particularly it should be understandable by ENT doctors, being its topic. However, the fact is that some paragraphs, and concepts, which are highlighted below, may be difficult to understand by readers (including the ENT) not too familiar with the vestibular physiology and its physiopathology. These passages should be corrected to ameliorate their comprehension.

We thank the reviewer for their helpful feedback. We have revised the manuscript to address each of the reviewer’s comments and provided point-by-point responses below.

More precisely:

I would suggest a more detailed title: Restoring vestibular afferent dynamics in Macaque monkeys enhances sensorimotor performance: implications for vestibular prostheses in Human

We thank the reviewer for this suggestion and have accordingly revised the title to: Restoring vestibular afferent dynamics enhances sensorimotor performance in Macaque monkeys: implications for vestibular prosthesis use in humans

Introduction

The authors state that their protocols used "virtual head motion "in the two monkeys with complete vestibular loss. That is, the prosthesis pulses were delivered as if the monkeys' heads were moving, but they were actually stationary in the chair. Why was this option was chosen? It means that the afferent information encoded by the spindles of the neck muscles did not occur during these virtual head motion, which could have impacted the VOR since proprioceptive information converge with the vestibular information in the vestibular nuclei and reticular formation. That would plead to test the stimulations during real head movements. Why it was not done? Please comment that point for the reader.

To directly address the reviewers’ main concern, we have and added to the revised manuscript new data on the prosthetic stimulation patterns evoked by actual physical head movements. Specifically, we now show – for the same two animals - the eye movements that are evoked by prosthesis stimulation encoding real-time head velocity that was detected by the gyroscope module of the prosthesis. These additional data are shown in new figures fig. S4, S5, and S7. Overall, our results for the virtual-rotation and physical-rotation VOR conditions show similar trends for both sinusoidal rotations (compare Fig. 3 to Figs. S4-5; dark and light conditions, respectively) and transient rotations (compare Fig. 4 to Fig. S7). As is now described in the revised results, higher gains were indeed observed in the physical-rotation conditions at higher stimulation frequencies (>5Hz). Also, higher gains were observed in the physical-rotation versus virtual-rotation transient condition.

We also now explain our rationale for appling “virtual head motion” stimulation was to better isolate the vestibular contribution to the VOR in the revised Results and Discussion. As appreciated by the reviewer, the application of real head motion will activate muscle spindles and thus the brain will then also have access to proprioceptive information. Furthermore, in a prior study our group has shown that neck proprioceptive inputs are upweighted in the vestibular nuclei after bilateral vestibular loss (Sadeghi et al. 2012). Thus, we focused our analysis on the "virtual head motion" with only vestibular information (via the prosthetic stimulation) since the up-weighted contribution could complicate the interpretation of the results based on our short-term experiments.

*“The vestibular system detects our head motion relative to space. In turn, this information is used to generate essential stabilizing reflexes and to provide us with our subjective sense of motion and orientation.”* I would suggest a more detailed sentence to help the uninformed reader: *In turn, this information is used to generate essential stabilizing reflexes and complex motor synergies to control gaze and posture and to provide us with our subjective sense of motion and orientation.*

We have revised the text as suggested.

*First, the three axes of head rotation are encoded by three separate sensory organs (i.e., the semicircular canals),* I understand that sentence, but again it may be confusing for the uninformed reader not to mention the otoliths sensors in one way or the other at that stage.

We thank the reviewer for this suggestion and have revised the text as suggested to include descriptions of both canal and otolith sensors.

To address this question, here we directly tested whether implementing the natural response dynamics of vestibular afferents improves prosthesis performance.
Please add that this was done in a primate model

We thank the reviewer for this suggestion and have revised the text to reflect the suggested change. The sentence now reads “To address this question, here we directly tested whether implementing the natural response dynamics of vestibular afferents improves prosthesis performance in a nonhuman primate model.”

parameter space, Please define that concept

We have revised the Introduction to more clearly define parameter space.

*“Using computational methods, we then demonstrated that the same model could account for the eye movements evoked by both sinusoidal and transient stimulation, and that the efficacy between stimulation pulses and afferent firing is substantially less than 1:1 as often assumed.”*

This point is important, and the description is too sketchy to be easily understood by an average reader at that stage of the manuscript. If I get it well, do the authors mean that it is often assume that a single pulse of stimulation would trigger each time one (or several?) action potentials? If it is the case how a computational model can test that hypothesis and why an experiment approach was not used also.

We thank the reviewer for raising this question. Yes, the reviewer is correct that the common assumption is that each stimulation pulse will elicit an action potential in each afferent. We have revised the text to clarify this point. This sentence now reads “Using computational methods, we then demonstrated that the same model could explain the eye movements evoked by both sinusoidal and transient stimulation, and that a stimulation efficacy substantially less than 100% could account for our results — that is, each stimulation pulse does not always evoke an afferent action potential”

Additionally, we have revised the manuscript (Results and Discussion) to emphasize that we have both theoretical and experimental evidence to support this hypothesis.

- First, our computational model suggests that the common assumption is not valid. We speculate that for this reason, eye movements were evoked in a linear manner well beyond the firing rate range of the afferents (Figure 6B).

- Moreover, in revisiting results of our prior work we discovered that there is direct support for this proposal based on our previously published data set that used same vestibular prosthesis in rhesus monkeys (Mitchell et al., 2016). Notably, in our Mitchell et al. study (Nature Communications) we recorded directly from afferents during prosthetic stimulation, and showed that stimulation efficacy was only ~27% efficacy in monkey with comparable VOR eye movement gain as monkey G. Because we had not fully understood the implications of this important finding in our previous paper, it was never discussed in any context.

Methods

*“by two mappings mimicking the natural high-pass response dynamics of the two types of vestibular afferents.”* A more detailed explanation of the terms "natural high-pass response dynamics" is required for the reader unfamiliar with the vestibular neurophysiology and possibly a figure to illustrate that point also.

We thank the reviewer for this suggestion and have revised the methods. We now include a more detailed description of the afferent dynamics and include a reference to Fig.1, which illustrates these dynamics.

All surgical and experimental protocols were approved by the Johns Hopkins Animal Care and Use Committee.
Date of approval, reference of the demand and as stated by PNAS

Animal Research (involving vertebrate animals, embryos or tissues)
Provide the name of the Institutional Animal Care and Use Committee (IACUC) or other
relevant ethics board that reviewed the study protocol, and indicate whether they
approved this research or granted a formal waiver of ethical approval
* Include an approval number if one was obtained
* If the study involved non-human primates, add additional details about animal welfare
and steps taken to ameliorate suffering

We have added the details of animal welfare including housing, feeding, environmental enrichment, and steps taken to minimize suffering as stated by the PLOS Biology guidelines. Additionally, note that our protocol number is PR19M408, which is affective through 10/17/2022.

The vestibular function, the way it is written for the two monkey is confusing and need to be revised. Monkey Y was implanted with canal intact in the right ear. In addition, it had non-functioning old prosthesis electrodes in the left ear. Reading that description, this monkey should have an intact vestibular function. Why it ended up with a model of bilateral vestibular loss? Monkey G had received gentamicin treatment via bilateral intratympanic injection. Presumably this one should have a bilateral vestibular loss as a result of the injection. In that condition, why subsequently, a labyrinthectomy in the right ear was performed and why on one side only?

We have revised the methods to make the history and status of the animals clearer. Specifically, we now clarify that vestibular implantation disrupts the canal function, causing vestibular hypofunction in the implanted side.

- Monkey Y’s left ear had been first implanted prior to implanting the right ear. As a result, monkey Y had bilateral vestibular hypofunction (VOR gains of <0.1 as is stated).
- Monkey G had received bilateral gentamicin treatment via intratympanic injection. Because the VOR gain was not sufficiently reduced, we performed a labyrinthectomy on the right ear, and implanted the left. Again, because protheses implantation disrupts the canal function, the monkey had bilateral hypofunction and as a result had bilateral vestibular hypofunction (VOR gains of <0.1 as is stated).

Typo problems in my text : 170 uA and 250 uA. Presumably micro Amp ? digitized at 30 kHz and later synced: what mean syned? Synchronized

We thank the reviewer for pointing out the typos and have corrected them in the methods section.

**Reviewer #2:**

In this study, the authors investigate how to optimize vestibular prostheses (that restore the sense of rotation by stimulating the vestibular organs in the inner ear). They demonstrate that incorporating in the prosthesis a transfer function that mimics the vestibular afferents' dynamics improves the temporal accuracy of behavioural responses in two rhesus monkeys. This study is an important step in the development of neuroprotheses, and also an impressive technical achievement.
The main conclusion of the study (regarding the dynamics of the response) is solidly supported. However, one limitation of vestibular prostheses is that their response gains are very low, and it is the case in this study. The authors propose that this is due to a poor stimulation efficiency between the prosthesis and the afferent, but this conclusion isn't be well supported, and should be reconsidered.

We thank the reviewer for their helpful feedback. We have revised the manuscript to address each of the reviewer’s comments and provided point-by-point responses below.

Major remarks:
The author observe that the gain of the VOR evoked by the prosthesis is 28% in one animal and 4.5% in the other. To explain this, they propose that the stimulation efficiency (i.e. the frequency of the discharge of the vestibular afferent relative to the pulse rate of the prosthesis) is 28% or 4.5% respectively. This can explain the observed data, indeed. However, the authors don't consider any alternative hypothesis. One possible alternative is that central VOR pathways readapt following the vestibular lesion. Another alternative is that the prosthesis evokes synchronous firing that is less efficient at driving VOR. None of these hypothesis can be discounted based on the author's data. Therefore, the authors haven't demonstrated that stimulation efficiency is less than 1:1, and this conclusion should be removed from the abstract, introduction, etc.

We appreciate the reviewer’s comments regarding the two alternate hypotheses that could also explain the observed low VOR gain. We have rewritten the Abstract and Introduction to be more precise, and revised the Results and Discussion sections to more systematically consider that several factors likely contribute to our observed findings.

First, we more clearly emphasize (see revised Results, and Discussion) that we actually have direct support that stimulation efficiency is less than 1:1 from previously published work (Mitchell et al., 2016, Nature Communications). Notably, in this study we recorded directly from afferents during prosthetic stimulation, and showed that stimulation efficacy was ~ only 27% in monkey with comparable eye movement gain as monkey G. Because we had not fully understood the implications of this important finding in our previous paper, it was never not discussed in any context. Importantly, these published results are consistent with the experimental data in the present study that relationship between stimulation rate and evoked VOR remains linear up 500 pps (Fig. 6B), even though vestibular afferents only reach a maximum firing rate of 350-400 pps.

Second, we have included a figure (new fig. S9) in the revised Results section to directly consider the reviewer’s second alternative hypothesis. Indeed, we had previously demonstrated that prosthesis stimulation rapidly induces a decrease in the synaptic efficacy of afferent-vestibular nuclei neuron transmission, that in turn results in a decrease in the evoked VOR (17% decrease, Mitchell et al., 2016). As discussed in this previous paper, we speculate that prosthetic stimulation induces long-term depression at this synapse due to the synchrony evoked across the vestibular afferents population – such synchrony is not present during natural head motion, Accordingly, we have revised the results to include an additional model to account for this prosthetic induced depression. As can now be seen in Fig. S9, our simulation results showed higher stimulation efficacy of 33.7% for Monkey G and 5.4% for Monkey Y.

Third, we also now consider the reviewer’s first alternative hypothesis. As stated in the revised Discussion, following bilateral loss, it is an open question how the central pathways adapt to process vestibular signals, because the vestibular contribution cannot be measured in the animals without a prosthesis. On one hand, it is possible central mechanisms could increase the synaptic weighting of the afferent input as has been observed following unilateral peripheral loss (Sadeghi et al., 2010a, 2010b). On the other hand, it is possible that, in the case of bilateral peripheral loss, adaptive central mechanisms instead function to suppress the contribution from any residual vestibular afferent nerve input. Future experiments focused on distinguishing between these two possibilities can serve to inform the development of improved prothesis-based approaches to restore vestibular function. We consider these possibilities in the revised Discussion.

Note that this point has major implications for the development of vestibular prosthesis. If the low gain of the VOR is indeed attributable to a low stimulation efficiency, then the implication is that developing better stimulation methods could eventually restore normal VOR. On the other hand, if it is due to central adaptation (including possibly physiological changes in the vestibular nuclei), then this adaptation is the bottleneck.

As noted above, we agree and have revised the Discussion to consider the point that central adaptation may need to be mitigated to improve the prosthesis performance. Note that our collaborator, Dr. Gene Fridman, has been working on developing safe direct current stimulation as a potential alternative and or complementary approach to the pulsatile stimulation which could help mitigate issues regarding both low afferent stimulation efficiency and synchrony.

Figure 6 and S6: the gain of the response is much higher in animal G (28%) compared to Y (4.5%). The author show animal G in the main figure 6 and Y in the supplementary figure S2: this is not a very balanced way to present data. It feels like the authors emphasize animal G and, in layman language, 'hide the bad monkey' in the supplementary figure. Given how the figure is organized, there is all the space necessary to show both animals in Fig. 6, and no justification not to do so.

We thank the reviewer for this comment and have revised Fig 6 to illustrate data from both monkeys, and have updated the legend accordingly.

Figure 2A: superimposing the 'normalize eye velocity' to an 'inverted head velocity' is incorrect. First, the head is not actually moving so it would be proper to refer to a 'simulated' head velocity. Second, head velocity has a unit of degrees per second, whereas the normalized eye velocity is unitless. Third, and fundamentally, the authors are giving the impression that the VOR is approximately compensatory at 10Hz, which is not the case (Fig. S1). Merging Fig. S1 and Fig. 2 would be a more balanced way to present the results.

We thank the reviewer for this comment. We have revised Fig. 2 to now show the absolute gain which was previously shown in Figure S2. We have also revised the legend for Figs. 2 and 3 to address the reviewer’s concerns. Specifically, we clarify that dashed line indicates the simulated head velocity, scaled to facilitate phase response comparison.

Minor remarks:
Figure 3: when comparing Fig. 3A and Fig. 3C, it appears that the normalized gain of the VOR resembles a lot the gain of the afferent's transfer functions in Fig. 3A. In other words, in seems that the gain of the VOR compared to stimulation amplitude is constant. Given this, wouldn't it be sensible to program a transfer function with a flat gain, which would presumably induce a VOR with a constant gain along frequencies?

We appreciate the reviewer’s point regarding the VOR gain shape. To clarify, we would like to emphasize that the static mapping (Fig. 1B, gray) actually has a flat gain across all frequencies. This did not result in a flat VOR gain but showed a decrease in gain at high frequencies (5-20 Hz; Fig. 2B, gray). We have revised the Results to make this point clearer.

Methods: could you give more details about the animal's history? How much time elapsed between the implantations and the recordings shown here? Was the prosthetics continuously active in their home cage (or used on a regular basis)? Were they used in other studies? With the same implants?

We have revised the methods to make the history and status of the animals clearer.

Notably, studies were done 10 years after implantation for Monkey G and 2 years after implantation for Monkey Y. The prosthesis was only turned on acutely during experiments with 1 hour baseline adaptation time (see Dai et al., 2011) prior to the onset of experimental testing. Monkeys G and Y were also only tested with acuate stimulation—both with the same implant—in prior experimental studies of VOR 3D tuning.

**Reviewer #3:**

This is an excellent piece of scientific work in a challenging area. I have no major criticisms.

We thank the reviewer for their helpful feedback. We have revised the manuscript to address each of the reviewer’s comments and provided point-by-point responses below.

The absolute gain responses are relatively low as shown in the supplementary information. I think at least an insert of the absolute gains should be shown in one of the main figures to give an idea of the current state of play of vestibular prostheses.

We thank the reviewer for this comment and have revised Fig. 2 to now show the absolute gain which was previously shown in Figure S2.

Did the investigators at any point, give real inertial stimuli in the light with the vestibular prosthesis turned on? If the vestibular prosthesis is only ever switched on in the dark to generate virtual eye movements, then there is no opportunity for the system to calibrate (and adapt) itself to the artificial input, irrespective of how well the investigators attempt to emulate natural stimuli. Hence, have the authors assessed sinusoidal VOR in the light with prothesis on and off? If so, does the VOR gain improve over time?

We thank the reviewer for his/her comment.

1) First, to directly address this concern we have added new data showing the prosthetic stimulation patterns evoked by actual physical head movements, in both dark and light, to the revised manuscript. Specifically, we now show – for the same two animals - the eye movements that are evoked by prosthesis stimulation encoding real-time head velocity that was detected by the gyroscope module of the prosthesis. These additional data are shown in new figures fig. S4, S5, and S7. As can be seen in the revised manuscript, the trends are the same for the virtual-rotation and physical-rotation conditions. Also as is now described in the revised results: i) higher gains were observed in the physical-rotation conditions at higher stimulation frequencies (i.e., >5Hz) and ii) higher gains were observed in the physical-rotation versus virtual-rotation transient condition. Thus, together these results indicate that the observed improvement in VOR accuracy with more biomimetic mappings was consistent across movement conditions, and that the higher VOR gains could be achieved during physical head rotations.

2) Also, in response to the reviewer’s comment, we compared the gain and phase of the first and last four cycles of the VOR responses evoked by sinusoidal prosthetic stimulation patterns triggered by physical head movements, to see if there were adaptive changes in responses over time. This is now presented in the Results accompanying Figure 3, as well as new supplementary Figures S4&5. Overall, we found no significant difference for any stimulation frequencies across mappings, in either dark or light conditions (p>0.05, Wilcoxon rank sum test, Bonferroni corrected). In our future work, we plan to incorporate the longer-term adaption paradigms.

In this regard, the authors mention that "the vestibular system is uniquely suited for prosthesis development" with which I agree. But there is little mention in the introduction about the development of the prosthesis being a clinically useful device for patients. In that regard, the authors should mention that human patients generally adapt well to bilateral vestibular loss with exercise so the benefits of a vestibular prosthesis on top of vestibular exercises would need to be balanced by the risks of the procedure/device. This risk-benefit curve would tilt one way or the other depending upon the relative efficacy and safety of the prosthesis. Such an argument would be useful in explaining the need for the current animal-based work (to improve efficacy and lower risks).

We agree this is an important point for discussion and have now consider the risk-benefit tradeoff in our revised Discussion.

Has there been any finite element modelling to assess how the e-fields are affected by changing the stimulus parameters, which in turn may influence the impact upon local and not so local neural structures. I am not saying this needs to be done, simply it would be interesting to know if this has been done.

We thank the reviewer for raising this point. A previous paper from our group (Hedjoudje et al. 2019) investigated the effect of electrode pair selection and the non-specificity of the stimulation (i.e., current spread between the three rotational axes) that informed the electrode pair selection in the present study. We agree this is an important direction for future investigation regarding objective criteria for optimizing the prosthesis placement and now note this in the revised Discussion.

The assumptions and coefficients on pg 14 could only be easily understood by researchers who performed the studies that were referenced, but would otherwise require me to dig into the original papers. Is there anyway to justify the various parameter selections a little more without over-inflating the manuscript?

We have revised the methods section to make the parameter selection clearer. We now specify that all but one parameter was taken directly from previous literature (Robinson 1981). We also specify that the plant dynamics constant $T_{e2}$ was adjusted from 0.016 to 0.008 and 0.025 for Monkey Y and Monkey G, respectively, to match the low-pass dynamics of the oculomotor plant in each monkey (i.e., the time constant required to account for the different slopes seen in Fig. 6B).

Page 16 - "Pathway modeling. Overall, a linear systems based VOR pathway model (Glasauer, 2007;Robinson, 1981) was used to predict the eye velocity from the afferent firing rate:" Does this statement assume that the afferent firing rate is 1:1 with stimulus frequency (as presumably you are not measuring the afferent firing rate - see next comment).

We thank the review for raising this question. The reviewer is correct that in our modeling, we first assumed an efficacy of 1:1 and found that the model over-predicted the velocity (Figure 6D left). We next added an adjustable efficacy term to the existing model. Notably to fit the data, we were required to reduce this efficacy term from to 28% and 4.5%, respectively (Figure 6D right). Finally, in the revised manuscript we now run a third model that accounts for the adaption of the afferent-central vestibular nuclei neuron synapse to prothesis stimulation, which we previously observed experimentally (Mitchell et al. 2016). We have revised the Methods section to make the sequence of our modeling approach clearer.

But, I think an important finding is the implication of a 28% (or 4.5% for monkey Y) stimulus efficacy. If this is an important barrier for clinical implementation, can the authors comment about the potential to surmount this barrier?

Yes, we speculate that stimulus efficiency will likely be an important barrier to overcome to improve clinical implementation, and we have added a new paragraph to the end of the Discussion of the revised manuscript to considers this point. Note that our current work in this area is focused on developing physiological and anatomical based modeling of the afferents activation with the goal of developing the next generation prosthesis increase the efficacy. In addition, our collaborator, Dr. Gene Fridman, is working on a device based on safe direct current stimulation as an alternative approach to the pulsatile stimulation which could help mitigate issues regarding both low afferent stimulation efficiency and synchrony.

For the reported P values, can the mean and the statistic be consistently reported. Also, when relevant, correct the P value for multiple comparisons.

We thank the reviewer for this comment and have revised the Results to correct for multiple comparisons (Bonferroni) where applicable. In addition, we now report the mean and statistic in our description of significant differences observed in our analysis of the VOR responses to transient data. Note, we elected not to do this in the case of the sinusoidal condition since the Bode plots provided clearly show each value, at each frequency, for each mapping, for each monkey – and listing these values in the text would reduce readability.
