# Supplementary material for: A prosthesis utilizing natural vestibular encoding strategies improves sensorimotor performance in monkeys
Source: PLoS Biol. 2022 Sep 14;20(9):e3001798. doi: 10.1371/journal.pbio.3001798 (PMC9473632; doi:10.1371/journal.pbio.3001798)
Supplement: Attachment — Submitted filename: 2022 08 09 response.docx [file pbio.3001798.s011.docx]

Reviewer remarks:


Reviewer #1: The reviewer thanks the authors to have taken into account all his suggestions and in particular to have include new data on prosthetic stimulation patterns evoked by actual physical head movements.


Reviewer #2: The authors did a very nice job at revising an already impressive manuscript. I particularly appreciate the thoughtfulness with which they responded to my remarks, and I have no further comments to make.


Reviewer #3: I am satisfied with the author's replies and changes in the manuscript in response to my comments. The changes further strengthen the paper.

I have two small comments about the Discussion.

The paragraph in Discussion Ln 447 is important as it highlights how different stimulus settings may suite different vestibular mechanisms, e.g. VOR vs. perception vs. postural control, important for future optimisation of the prosthesis - as they note in Ln 456 - "Thus, we speculate that the biomimetic irregular mapping might be better suited for postural control and self-motion perception, whereas the biomimetic regular and mixed mappings produced the most temporally accurate VOR eye movements as was observed in our present study.", hence....

- the authors should make a note in their manuscript that in humans, cortical networks mediating vestibular perception and postural control actually overlap in the right temporal cortex in the inferior longitudinal fasciculus (Calzolari et al., Brain DOI: 10.1093/brain/awaa386). It follows that in line with their comments, future prosthesis optimisation should involve perceptual and postural measures.
- In Ln455 they should qualify the contribution of ascending pathways to perception a little more precisely. These pathways specifically convey the vestibular signals to cortical brain circuits and it is these brain circuits that are more closely engaged in the processes that mediate vestibular perception (add reference doi: Hadi et al. BioRxiv https://doi.org/10.1101/2021.12.03.471139). This seems a little pedantic since what the authors wrote was not wrong, but the suggested change seems clearer to my mind.

We thank the reviewer for their thoughtful comments on the Discussion. We have revised the Discussion (Ln453-457) to more precisely qualify the contribution of ascending pathways to cortical brain circuits mediating perception and also to note that these circuits overlap with brain areas mediating postural control in humans. We have also incorporated the suggested citations.
